# Total Nasal Reconstruction Redefined: Classic Craft Meets Microsurgical Innovation

**DOI:** 10.1097/SCS.0000000000012507

**Published:** 2026-02-02

**Authors:** Dariush Nikkhah, Petko Shtarbanov, Allan Ponniah

**Affiliations:** * University College London, London, United Kingdom; † Royal Free London NHS Foundation Trust, London, United Kingdom

**Keywords:** Flaps, medial femoral condyle, microsurgery, nasal reconstruction, prefabrication

## Abstract

The authors illustrate a unique case of total nasal reconstruction that successfully combined historical reconstructive techniques with modern microsurgery, supported by artificial intelligence-assisted operative planning. The patient presented with multiple failed nasal reconstructions, previously performed at different surgical units, aimed to correct congenital nasal dysplasia due to Binder syndrome. In this case, a prelaminated radial forearm free flap was used for the nasal lining, a costal cartilage tripod for structural support, and a delayed forehead flap for external coverage. To reconstruct the nasal septum and ensure an anatomically aligned airway, the authors employed a vascularised corticocancellous free flap from the medial femoral condyle. Fourteen months postoperatively, the patient had a well-healed, mucosalised airway and an improved nasal structure.

Sir Harold Gillies pioneered key techniques adopted today in reconstructive surgery. His tubed pedicle flap served to rebuild faces in the Great War.^1^ Although allotransplantation has moved the field of unreconstructible facial disfigurements, it poses a significant risk of rejection and poor long-term outcomes.^2^ We present a novel case of total nasal reconstruction that combined established techniques, described over a hundred years ago by Gillies, and modern microsurgical methods utilising autologous free tissue transplantation.

## CASE PRESENTATION

A 38-year-old woman presented to our surgical department with congenital nasal dysplasia secondary to Binder syndrome and a history of failed childhood nasal reconstructions using tissue expansion and nasolabial flaps. Our approach aimed to reconstruct the central face by providing durable internal lining, robust cartilaginous support, and a functional airway. Throughout the patient’s reconstructive course in our unit, three-dimensional image printing and artificial intelligence-predicted facial reconstruction models informed clinical planning and supported shared decision-making about final aesthetic and functional goals.

**FIGURE 1:**
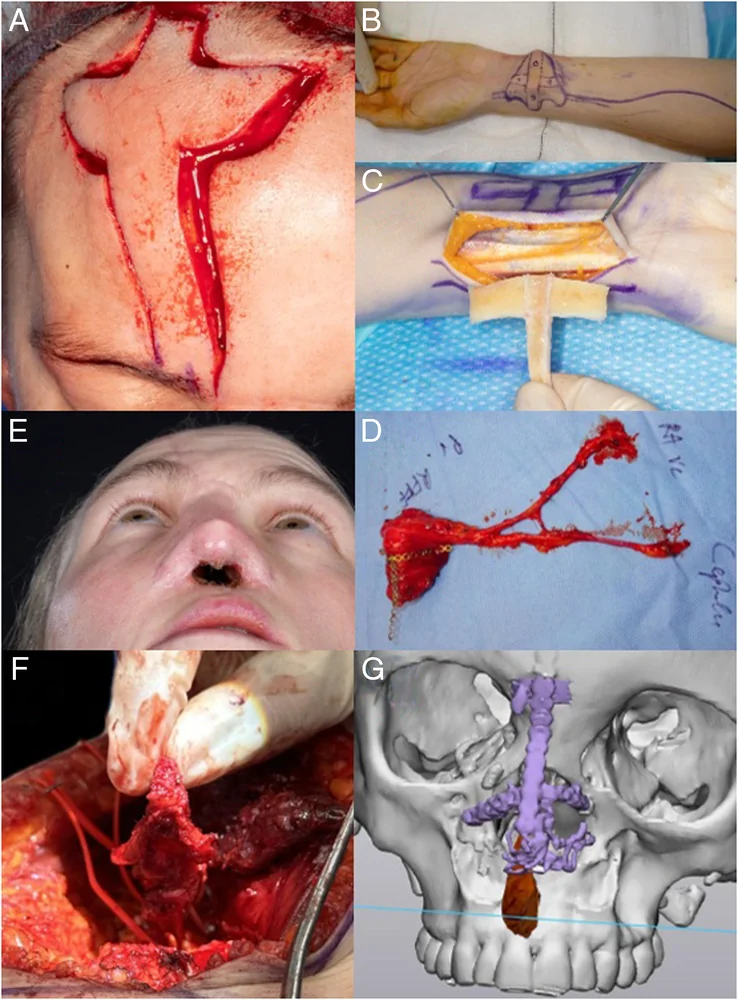
The delayed pedicled paramedian forehead flap was raised to provide external nasal coverage (A). Prelamination for nasal reconstruction using a costal cartilage graft for a supportive framework, shaped to form a tripod, and banked with the radial forearm flap (B and C). The subsequent free flap for internal lining comprised the costal cartilage wrapped by the inverted radial forearm flap with associated vascular pedicle of radial artery, venae comitantes and cephalic vein (D). The initial nasal framework lacked an adequate septum following collapse and reabsorption of the columella secondary to pressure necrosis (E). The nasal septum was reconstructed using the medial femoral condyle flap (F), following guidance from virtual surgical planning (G) software (craniofacial module of MIMICS, Materialize N.V., Leuven, Belgium).

With the supratrochlear vessels compromised from prior procedures, we delayed the pedicled forehead flap (Fig. 1A) and banked a cartilaginous framework in the forearm, an adaptation of the Gillies technique known as prelamination. Instead of using the forehead flap for prelamination, we prelaminated the microsurgical radial forearm flap (Fig. 1B). Costal cartilage, taken from the rib, was shaped into a tripod structure and placed flat to prevent extrusion (Fig. 1C). After 8 weeks, we harvested the radial forearm flap, turning it inside out to create the internal lining (Fig. 1D); to our knowledge, this was a novel design. Using a craniofacial plate, we achieved rigid fixation of the vascularised transplant onto the maxilla and frontal bone, while the delayed forehead flap provided external cover. Microsurgical anastomosis was performed to the right facial vessels. We subsequently performed forehead flap debulking over several operative sittings.

Regrettably, prior attempts at nasal dilation with nasal splints had resulted in necrosis of the reconstructed columella (Fig. 1E). We made a radical decision to rebuild the septum using a corticocancellous free flap with vascularised fascia from the knee. The medial femoral condyle (MFC) flap was harvested from the patient in the supine position using a standard approach as previously described.^3^ The flap was shaped to provide fascia for the nasal sill and recreate the anterior bony septum (Fig. 1F), and anastomosed to the left facial vessels. The careful modelling for this free flap was assisted by virtual surgical planning software (Fig. 1G). A full-thickness skin graft was harvested from the left postauricular area, fenestrated and applied to the columella, left lateral nasal ala and over the MFC pedicle in the nasolabial fold. To the best of our understanding, this was the first reported application of an MFC flap to reconstruct the nasal septum. Confirmation of vessel patency and flap perfusion following anastomosis was made with indocyanine green fluorescence angiography. With regard to the postoperative flap protocol, we provided hourly hand-held Doppler ultrasound monitoring of the arterial signal.

**FIGURE 2:**
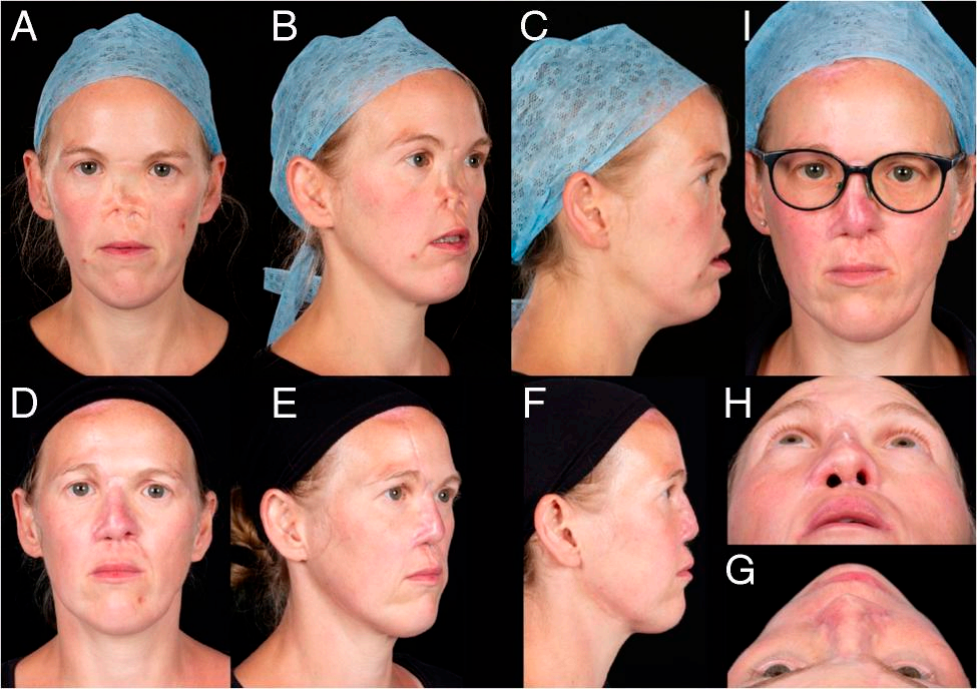
The patient presented to our surgical unit after multiple failed nasal reconstructive attempts in childhood (A–C). The results following total nasal reconstruction at 14 months postoperative follow-up are demonstrated in the following clinical photographs (D–I).

At 6 months postreconstruction, nasoendoscopy confirmed a well-healed, mucosalised airway without stenosis and minimal inflammation, allowing for functional breathing. Compared with the nasal structure at initial presentation (Fig. 2A-C), at 14 months postoperative follow-up, the overall outcome is aesthetically and functionally acceptable to the patient (Fig. 2D-I). Our patient can wear glasses for vision and uses fluticasone nasal spray as needed. Given the patient’s history of multiple operations before our interventions and a second free flap to the columella, there was a high risk of airway narrowing. Although they now tolerate several hours without nasal splints during the day, we believe scar remodelling can continue for up to 18 months, and thus ongoing splinting, particularly at night, is recommended to maintain airway patency. The techniques described herein, when used synergistically, enabled an outcome that represents an unprecedented achievement in nasal reconstruction—in the patient’s own words, the reconstructive surgery “opened a window in their face.”
